# Supplementary material for: Children’s descriptions of playing and learning as related processes
Source: PLoS One. 2020 Apr 15;15(4):e0230588. doi: 10.1371/journal.pone.0230588 (PMC7159209; doi:10.1371/journal.pone.0230588)
Supplement: Attachment — Submitted filename: Response to reviewers_final.docx [file pone.0230588.s001.docx]

Dear Dr. Sobel,

Thank you for submitting your manuscript to PLOS ONE. After careful consideration, we feel that it has merit but does not fully meet PLOS ONE’s publication criteria as it currently stands. Therefore, we invite you to submit a revised version of the manuscript that addresses the points raised during the review process.

First of all please accept my apologies for the delay on this decision. I have now received reviews from two expert reviewers. As you'll see, both reviewers are positively inclined towards this manuscript, and believe that it has the potential to make an important contribution to the field, as do I. I am generally in agreement with the reviewers' critiques, and most of their concerns are relatively minor. That said, each reviewer raises a more serious concern about the paper, both of which I shared. I want to highlight those concerns as particularly important to address if you revise this manuscript.

Reviewer 1 is troubled by the linguistic nature of the task, and is worried that the conclusion that children show "immature" understanding of playing and learning as processes may reflect task demands rather than a true lack of understanding. Reviewer 1 suggests that you might conduct a similar study using a non-linguistic task. I do not believe this is strictly necessary, but I would like to see a careful argument backing up the construct validity of the linguistic task, as well as a discussion of its limitations with respect to your conclusion.

Reviewer 2 suggests that the overarching question you are asking could be more strongly motivated. They note that some of this currently appears in the discussion, but should be highlighted in the introduction as well. In my own reading of the paper I do see the theoretical motivation for this work, but I agree that it could be more strongly laid out in the introduction.

I do hope you choose to revise this manuscript, as I think both these issues are addressable and can help clarify and solidify the contribution of this work. Please also be sure to attend to all of the more minor concerns each reviewer raises.

We would appreciate receiving your revised manuscript by Mar 02 2020 11:59PM. When you are ready to submit your revision, log on to <https://www.editorialmanager.com/pone/> and select the 'Submissions Needing Revision' folder to locate your manuscript file.

If you would like to make changes to your financial disclosure, please include your updated statement in your cover letter.

To enhance the reproducibility of your results, we recommend that if applicable you deposit your laboratory protocols in [protocols.io](http://protocols.io/), where a protocol can be assigned its own identifier (DOI) such that it can be cited independently in the future. For instructions see: <http://journals.plos.org/plosone/s/submission-guidelines#loc-laboratory-protocols>

Please include the following items when submitting your revised manuscript:

- A rebuttal letter that responds to each point raised by the academic editor and reviewer(s). This letter should be uploaded as separate file and labeled 'Response to Reviewers'.
- A marked-up copy of your manuscript that highlights changes made to the original version. This file should be uploaded as separate file and labeled 'Revised Manuscript with Track Changes'.
- An unmarked version of your revised paper without tracked changes. This file should be uploaded as separate file and labeled 'Manuscript'.

Please note while forming your response, if your article is accepted, you may have the opportunity to make the peer review history publicly available. The record will include editor decision letters (with reviews) and your responses to reviewer comments. If eligible, we will contact you to opt in or out.

We look forward to receiving your revised manuscript.

Kind regards,

Lucas Payne Butler
Academic Editor
PLOS ONE

Authors’ responses are in Red.

Journal Requirements:

When submitting your revision, we need you to address these additional requirements:

1. Please ensure that your manuscript meets PLOS ONE's style requirements, including those for file naming. The PLOS ONE style templates can be found at <http://www.plosone.org/attachments/PLOSOne_formatting_sample_main_body.pdf> and <http://www.plosone.org/attachments/PLOSOne_formatting_sample_title_authors_affiliations.pdf>

 We received errors when we tried to follow these links, but we believe that the manuscript is in the style requirement as per the PLOSOne website.

2. You indicated that you had ethical approval for your study. In your Methods section, please ensure you have also stated whether you obtained consent from parents or guardians of the minors included in the study or whether the research ethics committee or IRB specifically waived the need for their consent.

This is now added to the Procedures subsection of the Methods section.

 3. Thank you for stating the following in the Acknowledgments Section of your manuscript:

"This work was supported by the National Science Foundation (1223777 and 1420548 to DMS)."

We note that you have provided funding information that is not currently declared in your Funding Statement. However, funding information should not appear in the Acknowledgments section or other areas of your manuscript. We will only publish funding information present in the Funding Statement section of the online submission form.

We have removed funding information from the Acknowledgments.

Please remove any funding-related text from the manuscript and let us know how you would like to update your Funding Statement. Currently, your Funding Statement reads as follows:

"No. The funders had no role in study design, data collection and analysis, decision to publish, or preparation of the manuscript."

We have revised this statement to read: This research was funded by National Science Foundation 1223777 and 1420548 to DMS. The funders had no role in study design, data collection and analysis, decision to publish, or preparation of the manuscript.

4. We note that you have stated that you will provide repository information for your data at acceptance. Should your manuscript be accepted for publication, we will hold it until you provide the relevant accession numbers or DOIs necessary to access your data. If you wish to make changes to your Data Availability statement, please describe these changes in your cover letter and we will update your Data Availability statement to reflect the information you provide.

The DOI to access these data has been provided in the manuscript.


[Note: HTML markup is below. Please do not edit.]

Reviewers' comments:

Reviewer's Responses to Questions

**Comments to the Author**

1. Is the manuscript technically sound, and do the data support the conclusions?

The manuscript must describe a technically sound piece of scientific research with data that supports the conclusions. Experiments must have been conducted rigorously, with appropriate controls, replication, and sample sizes. The conclusions must be drawn appropriately based on the data presented.

Reviewer #1: Yes

Reviewer #2: Yes

2. Has the statistical analysis been performed appropriately and rigorously?

Reviewer #1: Yes

Reviewer #2: Yes

3. Have the authors made all data underlying the findings in their manuscript fully available?

The [PLOS Data policy](http://www.plosone.org/static/policies.action#sharing) requires authors to make all data underlying the findings described in their manuscript fully available without restriction, with rare exception (please refer to the Data Availability Statement in the manuscript PDF file). The data should be provided as part of the manuscript or its supporting information, or deposited to a public repository. For example, in addition to summary statistics, the data points behind means, medians and variance measures should be available. If there are restrictions on publicly sharing data—e.g. participant privacy or use of data from a third party—those must be specified.

Reviewer #1: No

Reviewer #2: Yes

De-identified data are available through a data repository. Identifiable data are not available, via our IRB review.

4. Is the manuscript presented in an intelligible fashion and written in standard English?

PLOS ONE does not copyedit accepted manuscripts, so the language in submitted articles must be clear, correct, and unambiguous. Any typographical or grammatical errors should be corrected at revision, so please note any specific errors here.

Reviewer #1: Yes

Reviewer #2: Yes

5. Review Comments to the Author

Please use the space provided to explain your answers to the questions above. You may also include additional comments for the author, including concerns about dual publication, research ethics, or publication ethics. (Please upload your review as an attachment if it exceeds 20,000 characters)

Reviewer #1: This manuscript explored children’s conceptual understanding of the relationship between playing and learning between the ages of 5-8-years-old. Children’s conceptual knowledge of playing and learning increased with age: they were more likely to define both terms as active processes and were also better able to generate examples of activities involving both playing and learning with age. Controlling for age, children with a more abstract metacognitive concept of learning were more likely to generate examples of activities involving playing and learning together. Children with a mature metacognitive understanding of playing and learning also were more likely to generate examples of playing with qualities of learning and vice versa. Thus, children’s understanding that play can lead to learning develops with age. Yet irrespective of age, conceptual knowledge of what it means to learn relates to children’s broader understanding of the relationship between play and learning.

Early childhood education has recently focused on play-based learning, yet past work suggests that young children think of play and learning as mutually exclusive. This paper adds to this literature by showing that children’s understanding of the relationship between play and learning develops with age. This work is a first step in a line of research identifying how children’s theories and beliefs about play and learning relate to their actual learning.

The main weakness of this paper is its use of a linguistic task to test conceptual change across development. The use of a linguistic task makes it unclear whether young children’s “immature” metacognitive understanding of learning and play stems from a true lack of conceptual knowledge or constraints on their expressive language ability. Thus, this paper would benefit from using a non-linguistic task, better arguments for their use of a linguistic task and its validity in young children, or stating this as a limitation in the discussion.

If the authors are able to address this major concern (as well as the minor ones laid out below), I believe this manuscript would be suitable for publication at Plos One and add valuable information to understanding the development of children’s conceptual knowledge of the relationship between playing and learning.

We have added a justification for the linguistic task and clarification that the length of children’s responses. (see Statistical Analysis section) We also calculated partial correlations controlling for the mean length of utterance for children’s definitions of playing and learning, to determine the effects of age over and above the effects of overall expressive language ability. Though this is a gross measure, we found age effects that remained significant even when controlling for the length of children’s answers (see Results section).

Abstract

• The authors should state their developmental change findings in the abstract.

We added a summary of the main developmental findings into the abstract.

Introduction
• Can the authors better define what would count as evidence for an abstract understanding of learning and playing? (last paragraph page 4).

Added a clarification here (now on p.5).

• Can the authors expand on their hypothesis that children who describe learning and playing as active processes might better be able to generate activities classified as both? How is this evidence specifically for metacognition? Couldn’t children also be using a simple heuristic that active processes share properties without really understanding how play and learning relate?
• Is there any evidence that representing play as learning relates to real world behavior in children?

If children believe the labels “playing” and “learning” only describe activities based on content, they might not think that there can be any overlap – if I am engaged in playing, then I am not learning, and vice-versa. If children see playing and learning as more abstract processes that involve thinking in particular ways, then they might see how playing and learning can happen concurrently. For example, if children believe that learning involves discovering something new, then that could happen while they are playing a game they have never played before (and thus learn something about the game). Similarly, if learning only means “math”, then it’s unlikely that children will believe that they can learn through play (or at least, it’s less likely that they can come up with examples of times when they have learned and played at the same time).

Method
• How was the sample size chosen? Do the authors have enough power to detect results?

Our analyses were mostly correlational in nature. To assume power = .8 and assuming alpha = .05 and a medium effect size in our analyses, we would need n=84 for the correlational analysis (via an a priori power analysis), but n=87 for the analysis at the end. We wound up with a sample of 92, which was a combination of our needing to fulfill our counterbalancing and overtesting on the final session at the museum (as all of our data came from museum-based testing). The significance levels of the analyses reported in the manuscript do not change if the last few participants are excluded. As a result, we decided to include the full dataset.

• I found it difficult to follow the analyses in the results section. The authors should add an analysis plan section to the methods to better guide the reader through the results. In the analysis plan, they should state any composite measures they will look at and which models they used and why.

We added an analysis plan to the Statistical Analysis section, and included information about how composite variables were calculated and what factors were included in each analysis. The results section is now broken down into two sections based on the analysis plan to make the findings easier to follow.

Results
• Why (and how) did the authors combine source, process, and outcome into a single factor? This is a bit strange since it means that process was looked at twice – within this factor and alone. It’s also confusing which outcome measures they looked at individually – I suggest the authors restate them all either here or in the suggested analysis plan section.

We have clarified our coding and analyses in the analysis plan. We simplified the analyses so that process-based definitions of playing now include information about who one played with (source), how one played (process), and/or the outcomes of playing (outcome), and these subcategories are not analyzed separately. We combined these categories into one factor because they most closely align with process-based definitions of learning in prior work (in which children focused on with whom or how they learned.

• I have some concern about the analyses in Table 1. Since the authors are predicting binary outcome measures, did they use logistic regressions? The authors should also correct for multiple comparisons since all the tests look at features of the same construct (within play or learning). Finally, the small sample sizes in some tests (e.g. No response in playing has an n = 3) render them underpowered. The authors may want to consider a different analysis approach or note this as a limitation.

We have eliminated these analyses, and only focused on the analysis of children’s metacognitive process definitions. We will point out that the analysis is on the entire sample, and is sufficiently powered (see note on sample size above).

• A graph of age effects would help the reader understand when kids start verbally endorsing a metacognitive/ process use of play and learning

We have added Figure 1, which contains this information.

• Does overall length of verbal responses increase with age?

We have added MLU to our analyses of children’s definitions of playing and learning (see Results, p. 13). The overall length does correlate with age, but we found that effects of age on the presence of metacognitive definitions remained significant even after controlling for MLU.

Discussion
• The authors write that “children were also more likely to state that their examples of play were also examples of learning (regardless of whether the attributes inherent in the activity related to learning) than to state that their examples of learning were also play.” What do adults do? It seems that adults would also be more likely to endorse that play leads to learning than vice versa. Is this a general bias we have or truth?

Though this study has not been done with adults (to our knowledge), we note in the introduction that adults often view play and learning as distinct from one another, and added a sentence to the discussion linking our findings to prior work on adults’ perceptions of play and learning. (p.19)

Reviewer #2: PONE-D-19-32302
Title: Children’s descriptions of playing and learning as related processes

The present study examined 5- to 8-year-olds responses to their definitions of playing and learning, and examined whether children defined them as active processes or by particular types of activities. This is an interesting study and the manuscript is well written.

The introduction provides a nice overview of the literature on children’s views of play, however, the authors should provide a stronger argument as to why they believe the research questions are important. That is, why is it important that we understand children’s perspectives on the distinction between play and learning? How is it relevant for their development or learning, or how children engage in a classroom? The authors do address the importance of the research questions in the discussion, but it is important for the authors to also discuss this in the introduction.

We have added more justification for the study in the introduction to describe why our research questions were important to answer, and to foreground the implications for educational practice.

It would be interesting to note what type of schooling the children attended (public schools, Montessori, etc.). It seems like this could influence children’s perspectives.

Though this is an interesting question, we did not collect data on the type of school children attended. We noted this limitation in the discussion.

Were gender and age stratified in the randomization of the two conditions?

No. Children were randomly assigned to one of the two conditions at the start of the task.

The authors should indicate the approximate length of the interviews with the children.

We added approximate length of the interviews to the Procedures section.

The analyses seem appropriate and the results section is well written.

Again, the discussion indicates why understanding children’s perspectives on play and learning is important to understand. It seems as though it would be better to include this in the introduction as well.

We added more detail about the implications of the study up front in the introduction to motivate the study.

6. PLOS authors have the option to publish the peer review history of their article ([what does this mean?](https://journals.plos.org/plosone/s/editorial-and-peer-review-process#loc-peer-review-history)). If published, this will include your full peer review and any attached files.


If you choose “no”, your identity will remain anonymous but your review may still be made public.


**Do you want your identity to be public for this peer review?** For information about this choice, including consent withdrawal, please see our [Privacy Policy](https://www.plos.org/privacy-policy).

Reviewer #1: No

Reviewer #2: No


[NOTE: If reviewer comments were submitted as an attachment file, they will be attached to this email and accessible via the submission site. Please log into your account, locate the manuscript record, and check for the action link "View Attachments". If this link does not appear, there are no attachment files to be viewed.]


While revising your submission, please upload your figure files to the Preflight Analysis and Conversion Engine (PACE) digital diagnostic tool, <https://pacev2.apexcovantage.com/>. PACE helps ensure that figures meet PLOS requirements. To use PACE, you must first register as a user. Registration is free. Then, login and navigate to the UPLOAD tab, where you will find detailed instructions on how to use the tool. If you encounter any issues or have any questions when using PACE, please email us at [figures@plos.org](mailto:figures@plos.org). Please note that Supporting Information files do not need this step.
